# Metagenome Sequencing to Explore Phylogenomics of Terrestrial Cyanobacteria

**DOI:** 10.1128/MRA.00258-21

**Published:** 2021-06-03

**Authors:** Ryan D. Ward, Jason E. Stajich, Jeffrey R. Johansen, Marcel Huntemann, Alicia Clum, Brian Foster, Bryce Foster, Simon Roux, Krishnaveni Palaniappan, Neha Varghese, Supratim Mukherjee, T. B. K. Reddy, Chris Daum, Alex Copeland, I.-M. A. Chen, Natalia N. Ivanova, Nikos C. Kyrpides, Nicole Shapiro, Emiley A. Eloe-Fadrosh, Nicole Pietrasiak

**Affiliations:** a Plant and Environmental Sciences Department, New Mexico State University, Las Cruces, New Mexico, USA; b Laboratory of Genetics, University of Wisconsin—Madison, Madison, Wisconsin, USA; c Department of Microbiology and Plant Pathology, University of California, Riverside, Riverside, California, USA; d Institute for Integrative Genome Biology, University of California, Riverside, Riverside, California, USA; e Department of Biology, John Carroll University, University Heights, Ohio, USA; f Department of Energy Joint Genome Institute, Berkeley, California, USA; University of Southern California

## Abstract

Cyanobacteria are ubiquitous microorganisms with crucial ecosystem functions, yet most knowledge of their biology relates to aquatic taxa. We have constructed metagenomes for 50 taxonomically well-characterized terrestrial cyanobacterial cultures. These data will support phylogenomic studies of evolutionary relationships and gene content among these unique algae and their aquatic relatives.

## ANNOUNCEMENT

Cyanobacteria, or blue-green algae, are found in nearly all aquatic and terrestrial habitats exposed to sunlight, ranging from marine systems, freshwater bodies, and thermal springs to soils and rock surfaces. They represent important ecosystem components, which perform crucial roles in biogeochemical cycling. However, knowledge of their taxonomy, phylogenomics, and physiology has been largely based on aquatic taxa. In the past 20 years, our team has discovered and described terrestrial cyanobacteria collected from extreme environments, including desert soils, ephemerally wet rock walls, and tropical damp cave walls, greatly enriching our understanding of cyanobacterial systematics (1–15). Reference strains of genus and species types are maintained in two algae culture collections at John Carroll University (JCU) and New Mexico State University (NMSU). The cultures are nonaxenic and represent unialgal polycultures containing primarily cyanobacteria and heterotrophic microbial associates. We used shotgun metagenomics of selected strains to support future investigation of the phylogenomic relationships of these unique algae.

**FIG 1:**
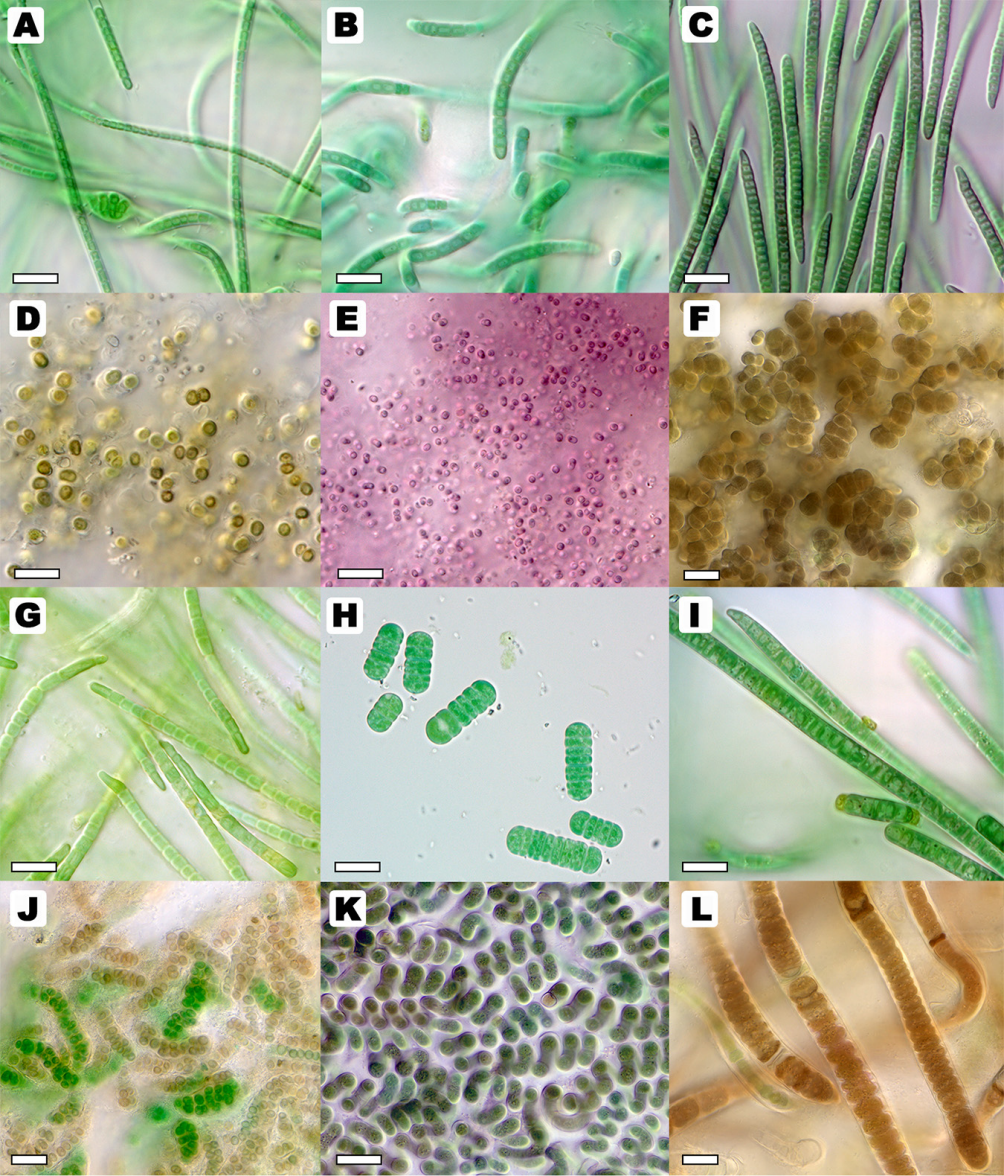
Light micrographs of representative cyanobacterial species maintained in unialgal cultures and studied in this project. Images represent cell morphologies in the stationary phase of the cyanobacterial life cycle. Wet mounts of cyanobacterial biomass were observed using a Zeiss AxioImager.A2 microscope equipped with Nomarski differential interference contrast optics and a Zeiss Axiocam 305 color camera at ×400 or ×1,000 magnification. (A) *Nodosilinea* sp. strain WJT8-NPBG4; (B) Myxacorys chilensis ATA2-1-KO14; (C) Trichocoleus desertorum ATA4-8-CV12; (D) Cyanosarcina radialis HA8281-LM2; (E) Aphanocapsa lilacina HA4352-LM1; (F) Pleurocapsa minor HA4340-MV1; (G) Kastovskya adunca ATA6-11-RM4; (H) *Hormoscilla* sp. strain CMT-3BRIN-NPC48; (I) Microcoleus vaginatus WJT46-NPBG5; (J) Mojavia pulchra JT2-VF2; (K) Spirirestis rafaelensis WJT71-NPBG6; (L) Brasilonema octagenarum HA4186-MV1. Bars, 10 μm (A–E, G–I, and K) and 20 μm (F, J, and L).

Fifty unialgal polycultures of cyanobacterial reference strains from the JCU and NMSU culture collections were selected, representing species taxonomically evaluated within the last 2 decades (Fig. 1). Detailed protocols for biomass growth, tissue harvesting, and DNA extraction can be found at protocols.io (dx.doi.org/10.17504/protocols.io.brg4m3yw). Briefly, biomass of each taxon was grown in liquid Z8 medium (16) and harvested after several weeks to months depending on the growth rate. Harvesting included cleaning, biomass concentration, biomass flash-freezing in liquid nitrogen, and storage at −80°C. We extracted DNA using the Qiagen DNeasy PowerLyzer microbial kit. The extraction procedure included an initial bead-beating step in a Precellys homogenizer for 45 s at 5,000 rpm, repeating four times. Samples were then processed following the manufacturer’s protocol for the kit and stored at −20°C until mailing to the Joint Genome Institute (JGI).

Plate-based DNA library preparation for Illumina sequencing was performed using KAPA Biosystems high-throughput library preparation kit p/n KK8235 on a PerkinElmer Sciclone next-generation sequencing (NGS) robotic liquid handling system. Then, 200 ng of sample DNA was sheared to 300 bp using a Covaris LE220 focused ultrasonicator. The sheared DNA fragments were size selected by double solid-phase reversible immobilization (SPRI), and then the selected fragments were end repaired, A tailed, and ligated with Illumina-compatible sequencing adaptors from IDT containing a unique molecular index barcode for each sample library. The prepared libraries were quantified with a KAPA Biosystems quantitative PCR (qPCR) kit on a Roche LightCycler 480 real-time PCR instrument. Genomic libraries were sequenced with a NovaSeq instrument (Illumina, San Diego, CA) using NovaSeq XP V1 reagent kits and an S4 flow cell following a 2 × 150-bp indexed run recipe. Demultiplexed reads were processed with BBDuk v38.87 (17) to remove contaminants, trim adapter sequence and “G” homopolymers ≥5 in size at the ends, quality trim reads, and remove reads with ≥4 “N” bases, with an average quality score of <3, or with a length of <51 bp. After processing, 2,021,674,638 reads remained (per sample range, 24,625,170 to 59,843,005; mean ± standard deviation, 40,433,493 ± 8,547,361).

Cleaned reads were assembled with metaSPAdes v3.13.0 (18) (-meta option) into contigs with parameters for a minimum contig length of 2 kb (-m 2000 option) and default kmer options for paired 150-bp reads. Across all assemblies, 745,331 metagenomic contigs were retained after quality control using AAFTF vecscreen v0.2.3 (19) (range of assembly GC content, 40.09% to 66.57%; GC content mean ± standard deviation, 59.94% ± 4.96%; range of assembly *N*_50_, 10,169 to 456,261 bp; *N*_50_ mean ± standard deviation, 133,466 ± 100,540 bp).

### Data availability.

All reads and assemblies were deposited under the NCBI accession numbers listed in Table 1. We also provided accession identifiers linking to JGI projects associated with each sample, including available IMG (20) data (Table 1).

**TABLE 1:** Metadata, accession numbers, and metagenome statistics for the 50 cyanobacterial cultures investigated in this study^*a*^

Species	Available at culture collections	Strain ID	Alternative strain ID	BioProject no.	SRA run no.	JGI IMG^*b*^	No. of reads	No. of contigs	*N*_50_ (bp)	GC content (%)	Habitat	Location
Aetokthonos hydrillicola	NMSU, JCU, CCALA	B3-Florida	CCALA 1050	PRJNA617093	SRR12347192	3300036376	56,817,878	54,864	184,060	63.45	Aquatic epiphyte	South Carolina, USA
Aphanocapsa lilacina	NMSU, JCU	HA4352-LM1		PRJNA677053	SRR13206895	3300039303	58,991,043	38,358	112,017	63.64	Cave rock wall	Hawaii, USA
*Aphanocapsa* sp.	NMSU, JCU	GSE-SYN-MK-11-07L		PRJNA621664	SRR11678123	3300035009	26,644,370	1,052	87,507	48.55	Desert wet wall	Utah, USA
Aphanothece saxicola	NMSU, JCU	GSE-SYN-MK-01-06B		PRJNA621658	SRR11676827	3300035003	50,225,611	8,874	272,324	66.57	Desert wet wall	Utah, USA
*Aphanothece* sp.	NMSU, JCU	CMT-3BRIN-NPC111		PRJNA617094	SRR12347633	3300036407	41,058,001	13,407	164,105	59.69	Desert soil	California, USA
Brasilonema angustatum	NMSU, JCU	HA4187-MV1		PRJNA617095	SRR12347719	3300036389	51,374,669	22,657	78,161	54.18	Tropical soil	Hawaii, USA
Brasilonema octagenarum	NMSU, JCU	HA4186-MV1		PRJNA621669	SRR11678155	3300035014	41,935,410	2,727	183,890	63.68	On wood in tropics	Hawaii, USA
*Calothrix* sp.	NMSU, JCU	FI2-JRJ7		PRJNA617096	SRR12347730	3300036390	26,844,447	1,488	168,980	56.22	Desert soil	California, USA
*Chroococcus* sp.	NMSU, JCU	CMT-3BRIN-NPC107		PRJNA653492	SRR12688413	3300037833	47,424,890	59,802	35,531	63.26	Desert soil	California, USA
Cyanomargarita calcarea	NMSU, JCU	GSE-NOS-MK-12-04C		PRJNA621661	SRR11676926	3300035006	30,710,984	5,791	264,901	58.92	Desert wet wall	Utah, USA
Cyanosarcina radialis	NMSU, JCU	HA8281-LM2		PRJNA653493	SRR12688412	3300037834	39,248,498	48,900	35,006	63.37	Cave rock wall	Hawaii, USA
Desmonostoc geniculatum	NMSU, JCU	HA4340-LM1		PRJNA621655	SRR11676647	3300035000	51,944,514	29,336	130,707	61.24	Cave rock wall	Hawaii, USA
Desmonostoc vinosum	NMSU, JCU	HA7617-LM4		PRJNA650882	SRR12951526	3300038554	36,690,552	6,487	166,368	60.96	Cave rock wall	Hawaii, USA
Drouetiella hepatica	NMSU, JCU	UHER 2000/2452		PRJNA617097	SRR12347860	3300036391	42,783,418	20,061	28,986	65.05	Rock surface	Kosice, Slovakia
*Gloeocapsa* sp.	NMSU, JCU	UFS-A4-WI-NPMV-4B04		PRJNA617098	SRR12347921	3300036443	29,499,512	17,169	32,766	60.53	Desert soil	Utah, USA
Goleter apudmare	NMSU, JCU, CCALA	HA4340-LM2	CCALA 1075	PRJNA621659	SRR11676930	3300035004	36,243,261	714	190,367	59.38	Cave rock wall	Hawaii, USA
*Hassallia* sp.	NMSU, JCU	WJT32-NPBG1		PRJNA617099	SRR12347922	3300036392	33,287,445	4,616	21,632	58.06	Desert soil	California, USA
*Hormoscilla* sp.	NMSU, JCU	CMT-3BRIN-NPC48		PRJNA650881	SRR12951525	3300038553	42,048,713	5,825	143,678	59.26	Desert soil	California, USA
*Iphinoe* sp.	NMSU, JCU	HA4291-MV1		PRJNA617100	SRR12349226	3300036393	31,352,639	32,008	107,621	62.85	Tropical soil	Hawaii, USA
Kaiparowitsia implicata	NMSU, JCU	GSE-PSE-MK54-09C		PRJNA650883	SRR13242888	3300038555	43,375,246	4,576	456,261	63.35	Desert wet wall	Utah, USA
Kastovskya adunca	NMSU, JCU, CCALA	ATA6-11-RM4	CCALA 1025	PRJNA621676	SRR11678224	3300034631	42,257,751	47,939	14,667	63.07	Desert soil	Atacama, Chile
Komarekiella atlantica	NMSU, JCU	HA4396-MV6		PRJNA650825	SRR12951531	3300038556	39,748,068	24,886	121,785	64.40	Tropical vernal pool	Hawaii, USA
*Lyngbya* sp.	NMSU, JCU	HA4199-MV5		PRJNA621654	SRR11676618	3300034630	37,379,750	5,629	214,210	61.23	Tropical soil	Hawaii, USA
Microcoleus vaginatus	NMSU, JCU	WJT46-NPBG5		PRJNA677056	SRR13207170	3300039305	40,531,812	28,602	165,791	60.22	Desert soil	California, USA
Mojavia pulchra	NMSU, JCU, CCALA	JT2-VF2	CCALA 691	PRJNA650826	SRR12951532	3300038557	54,924,618	10,021	208,763	62.75	Desert soil	California, USA
Myxacorys californica	NMSU, JCU, UTEX	WJT36-NPBG1	UTEX B 3157	PRJNA621674	SRR11678221	3300035019	32,998,795	9,403	81,746	64.17	Desert soil	California, USA
Myxacorys chilensis	NMSU, JCU, UTEX	ATA2-1-KO14	UTEX B 3158	PRJNA621667	SRR11678144	3300035012	46,028,284	19,551	69,559	60.50	Desert soil	Coquimbo, Chile
*Nodosilinea* sp.	NMSU, JCU	WJT8-NPBG4		PRJNA621656	SRR11676645	3300035001	36,650,447	2,944	173,054	65.12	Desert soil	California, USA
Nostoc desertorum	NMSU, JCU, CCALA	CM1-VF14	CCALA 693	PRJNA617101	SRR12349250	3300036444	55,466,988	7,058	211,685	62.42	Desert soil	California, USA
Nostoc indistinguendum	NMSU, JCU, CCALA	CM1-VF10	CCALA 692	PRJNA621666	SRR11678133	3300035011	34,734,084	13,226	98,409	54.74	Desert soil	California, USA
Oscillatoria princeps	NMSU	RMCB-10		PRJNA621670	SRR11678154	3300035015	35,756,581	29,084	28,454	61.51	Surface water	Lower Austria, Austria
Oscillatoria tanganyikae	NMSU, JCU	FI6-MK23		PRJNA621665	SRR11678132	3300035010	41,220,548	7,027	306,031	59.66	Desert soil	California, USA
Pegethrix bostrychoides	NMSU, JCU	GSE-TBD4-15B		PRJNA617102	SRR12349533	3300036394	34,506,500	2,385	397,454	61.39%	Desert wet wall	Utah, USA
Pelatocladus maniniholoensis	NMSU, JCU	HA4357-MV3		PRJNA653494	SRR12687981	3300037800	30,851,680	553	52,911	40.09%	Cave rock wall	Hawaii, USA
*Plectolyngbya* sp.	NMSU, JCU	WJT66-NPBG17		PRJNA617103	SRR12349534	3300036395	24,625,170	9,370	10,169	53.14	Desert soil	California, USA
Pleurocapsa minor	NMSU, JCU	GSE-CHR-MK-17-07R		PRJNA677054	SRR13207074	3300039304	44,764,195	25,094	40,205	64.01	Desert wet wall	Utah, USA
*Pleurocapsa minor*	NMSU, JCU	HA4230-MV1		PRJNA617104	SRR12349680	3300036396	59,843,005	708	316,355	63.57	Tropical rock wall	Hawaii, USA
*Scytolyngbya* sp.	NMSU, JCU	HA4215-MV1		PRJNA617105	SRR12349737	3300036104	33,289,893	8,107	243,918	60.55	Freshwater stream	Hawaii, USA
Scytonema hyalinum	NMSU, JCU	WJT4-NPBG1		PRJNA617106	SRR12349874	3300036520	40,826,558	35,954	57,285	64.29	Desert soil	California, USA
Scytonematopsis contorta	NMSU, JCU, UTEX	HA4267-MV1	UTEX 2964	PRJNA653495	SRR12688486	3300037859	41,139,075	2,560	124,805	50.66	Tropical vernal pool	Hawaii, USA
Spirirestis rafaelensis	NMSU, JCU	WJT71-NPBG6		PRJNA683097	SRR13242887	3300042538	30,810,560	12,769	49,002	61.71	Desert soil	California, USA
Stenomitos rutilans	NMSU, JCU	HA7619-LM2		PRJNA617107	SRR12350322	3300036446	39,342,385	29,421	125,980	59.27	Cave rock wall	Hawaii, USA
Symplocastrum torsivum	NMSU, JCU, CCALA, UTEX	CPER-KK1	CCALA 1031 UTEX B 3163	PRJNA617108	SRR12350505	3300036521	45,589,224	7,498	188,807	61.87	Grassland soil	Colorado, USA
Tildeniella nuda	NMSU, JCU	ZEHNDER 1965/U140		PRJNA621657	SRR11676687	3300035002	43,732,034	26,865	163,389	64.07	Temperate wet wall	Zurich, Switzerland
Tildeniella torsiva	NMSU, JCU	UHER 1998/13D		PRJNA653496	SRR12689282	3300037860	51,170,286	22,112	16,291	50.54	Temperate wet wall	Kosice, Slovakia
Timaviella obliquedivisa	NMSU, JCU	GSE-PSE-MK23-08B		PRJNA621673	SRR11678220	3300035018	43,160,626	11,520	86,031	58.41	Desert wet wall	Utah, USA
Tolypothrix brevis	NMSU, JCU	GSE-NOS-MK-07-07A		PRJNA621662	SRR11678125	3300035007	36,087,578	9,013	171,611	59.37	Desert wet wall	Utah, USA
Tolypothrix carrinoi	NMSU, JCU	HA7290-LM1		PRJNA621663	SRR11678124	3300035008	36,281,317	43,124	40,599	59.34	Cave rock wall	Hawaii, USA
Trichocoleus desertorum	NMSU, JCU	ATA4-8-CV12		PRJNA621668	SRR11678143	3300035013	37,070,905	5,401	91,542	60.17	Desert soil	Atacama, Chile
“*Trichormus*” sp.	NMSU, JCU	ATA11-4-KO1		PRJNA621660	SRR11676933	3300035005	32,384,820	832	238,030	52.69	Desert soil	Tarapaca, Chile

a Cyanobacterial cultures are available upon request from the research culture collections of Jeffrey R. Johansen (johansen@jcu.edu) at John Carroll University (JCU) and Nicole Pietrasiak (npietras@nmsu.edu) at New Mexico State University (NMSU). The selected strains are also available publicly at the University of Texas Culture Collection of Algae (UTEX) and the Culture Collection of Autotrophic Organisms in Třeboň, Czech Republic (CCALA). Accordingly, alternative UTEX and CCALA strain identifiers are given in the table.

b JGI IMG/M, Joint Genome Institute Integrated Microbial Genomes and Microbiomes database.

## References

[1] Řeháková K, Johansen JR, Casamatta DA, Xuesong L, Vincent J. 2007. Morphological and molecular characterization of selected desert soil cyanobacteria: three species new to science including *Mojavia pulchra* gen. et sp. nov. Phycologia 46:481–502. doi:10.2216/06-92.1.

[2] Mühlsteinová R, Johansen JR, Pietrasiak N, Martin MP, Osorio-Santos K, Warren SD. 2014. Polyphasic characterization of *Trichocoleus desertorum* sp. nov. (Pseudanabaenales, Cyanobacteria) from desert soils and phylogenetic placement of the genus *Trichocoleus*. Phytotaxa 163:241–261. doi:10.11646/phytotaxa.163.5.1.

[3] Mühlsteinová R, Johansen JR, Pietrasiak N, Martin MP. 2014. Polyphasic characterization of *Kastovskya adunca* gen. nov. et comb. nov. (Cyanobacteria: Oscillatoriales), from desert soils of the Atacama Desert, Chile. Phytotaxa 163:216–228. doi:10.11646/phytotaxa.163.4.2.

[4] Osorio-Santos K, Pietrasiak N, Bohunická M, Miscoe LH, Kováčik L, Martin MP, Johansen JR. 2014. Seven new species of *Oculatella* (Pseudanabaenales, Cyanobacteria): taxonomically recognizing cryptic diversification. Eur J Phycol 49:450–470. doi:10.1080/09670262.2014.976843.

[5] Patzelt DJ, Hodač L, Friedl T, Pietrasiak N, Johansen JR. 2014. Biodiversity of soil cyanobacteria in the hyper-arid Atacama Desert, Chile. J Phycol 50:698–710. doi:10.1111/jpy.12196. 26988453

[6] Pietrasiak N, Mühlsteinová R, Siegesmund MA, Johansen JR. 2014. Phylogenetic placement of *Symplocastrum* (Phormidiaceae, Cyanophyceae) with a new combination *S. californicum* and two new species: *S. flechtnerae* and *S. torsivum*. Phycologia 53:529–541. doi:10.2216/14-029.1.

[7] Bohunická M, Pietrasiak N, Johansen JR, Gómez EB, Hauer T, Gaysina LA, Lukešová A. 2015. *Roholtiella*, gen. nov. (Nostocales, Cyanobacteria): a tapering and branching cyanobacteria of the family Nostocaceae. Phytotaxa 197:84–103. doi:10.11646/phytotaxa.197.2.2.

[8] Miscoe LH, Johansen JR, Vaccarino MA, Pietrasiak N, Sherwood AR. 2016. Novel cyanobacteria from caves on Kauai, Hawaii, p 75–152. *In* Miscoe LH, Johansen JR, Kociolek JP, Lowe RL, Vaccarino MA, Pietrasiak N, Sherwood AR (ed), The diatom flora and cyanobacteria from caves on Kauai, Hawaii. Bibliotheca Phycologica, Stuttgart, Germany.

[9] Johansen JR, Mareš J, Pietrasiak N, Bohunická M, Zima J Jr, Štenclová L, Hauer T. 2017. Highly divergent 16S rRNA sequences in ribosomal operons of *Scytonema hyalinum* (Cyanobacteria). PLoS One 12:e0186393. doi:10.1371/journal.pone.0186393. 29073157 PMC5658200

[10] Shalygin S, Shalygina R, Johansen JR, Pietrasiak N, Berrendero Gómez E, Bohunická M, Mareš J, Sheil CA. 2017. *Cyanomargarita* gen. nov. (Nostocales, Cyanobacteria): convergent evolution resulting in a cryptic genus. J Phycol 53:762–777. doi:10.1111/jpy.12542. 28403525

[11] Mai T, Johansen JR, Pietrasiak N, Bohunická M, Martin MP. 2018. Revision of the Synechococcales (Cyanobacteria) through recognition of four families including Oculatellaceae fam. nov. and Trichocoleaceae fam. nov. and six new genera containing 14 species. Phytotaxa 365:1–59. doi:10.11646/phytotaxa.365.1.1.

[12] Pietrasiak N, Osorio-Santos K, Shalygin S, Martin MP, Johansen JR. 2019. When is a lineage a species? A case study in *Myxacorys* gen. nov. (Synechococcales: Cyanobacteria) with the description of two new species from the Americas. J Phycol 55:976–996. doi:10.1111/jpy.12897. 31233617

[13] Shalygin S, Kavulic KJ, Pietrasiak N, Bohunická M, Vaccarino MA, Chesarino NM, Johansen JR. 2019. Neotypification of *Pleurocapsa fuliginosa* and epitypification of *P. minor* (Pleurocapsales): resolving a polyphyletic cyanobacterial genus. Phytotaxa 392:245–263. doi:10.11646/phytotaxa.392.4.1.

[14] Mesfin M, Johansen JR, Pietrasiak N, Baldarelli LM. 2020. *Nostoc oromo* sp. nov. (Nostocales, Cyanophyceae) from Ethiopia: a new species based on morphological and molecular evidence. Phytotaxa 433:81–93. doi:10.11646/phytotaxa.433.2.1.

[15] Pietrasiak N, Reeve S, Osorio-Santos K, Lipson D, Johansen JR. 2021. *Trichotorquatus* gen. nov.: a new genus of soil cyanobacteria discovered from American drylands. J Phycol. doi:10.1111/jpy.13147. 33583028

[16] Carmichael WW. 1986. Isolation, culture and toxicity testing of toxic freshwater cyanobacteria (blue-green algae), p 1249–1262. *In* Shilov V (ed), Fundamental research in homogenous catalysis. Gordon & Breach, New York, NY.

[17] Bushnell B. 2020. BBMap. http://sourceforge.net/projects/bbmap/.

[18] Nurk S, Meleshko D, Korobeynikov A, Pevzner PA. 2017. metaSPAdes: a new versatile metagenomic assembler. Genome Res 27:824–834. doi:10.1101/gr.213959.116. 28298430 PMC5411777

[19] Stajich JE, Palmer J. AAFTF: v0.2.3: automatic assembly for the Fungi. 2019. doi:10.5281/zenodo.1620526.

[20] Chen I-MA, Chu K, Palaniappan K, Ratner A, Huang J, Huntemann M, Hajek P, Ritter S, Varghese N, Seshadri R, Roux S, Woyke T, Eloe-Fadrosh EA, Ivanova NN, Kyrpides NC. 2021. The IMG/M data management and analysis system v.6.0: new tools and advanced capabilities. Nucleic Acids Res 49:D751–D763. doi:10.1093/nar/gkaa939. 33119741 PMC7778900

